# Lessons learned for postoperative wound healing: respect the past and embrace the future

**DOI:** 10.1186/s13037-019-0185-5

**Published:** 2019-01-24

**Authors:** Boris A. Zelle, Philip F. Stahel

**Affiliations:** 1 Department of Orthopaedics, University of Texas Health, 7703 Floyd Curl Dr.,MC-7774, San Antonio, TX 78229 USA; 2 Rocky Vista University, College of Osteopathic Medicine, Parker, CO 80134 USA

**Keywords:** Patient safety in surgery, Surgical site infection, Wound healing, Complications

The field of patient safety in surgery has seen dramatic improvements in the new millennium [1]. Ongoing global initiatives keep a focus on assuring timely access to appropriate care for disparities while avoiding “low value” surgical care with the goal of optimizing long-term patient outcomes [2, 3]. The widespread implementation of fast-track surgery through evidence-based “enhanced recovery after surgery” (ERAS) programs has improved key outcome metrics on a global scale, including shorter hospital length of stay, reduction of opiate use for perioperative pain control, increased patient experience scores and improved outcomes [4]. In addition, the widespread implementation of standardized sepsis intervention bundles for early recognition and treatment of patients at risk of adverse outcomes from sepsis has led to a dramatic decrease of mortality and morbidity in highly vulnerable patient populations [5]. Notwithstanding the impressive achievements by proven best practices and modern evidence-based approaches for surgical care, complications related to postoperative wound healing remain unresolved conundrum in the twenty-first century [6–10]. From a historic perspective, there have been multiple landmark achievements in the arena of aseptic surgical technique [11]. The revolutionary “germ theory”, postulated by Louis Pasteur in the 1850s, was followed by pivotal improvements in the field of antiseptic techniques, such as the use of phenol as a disinfectant spray (Lister in 1865), steam sterilization (Bergmann in 1891), rubber gloves (Halstead 1890), and surgical masks (Mickulicz-Radecki 1897) [12, 13]. The subsequent groundbreaking discovery of penicillin by Sir Alexander Fleming in 1928 further laid the foundation for the administration of perioperative antibiotic prophylaxis and represented a foremost contribution to the development of safe surgical techniques [14]. The implementation of antiseptic surgical techniques also opened the door for the development of surgical implants, which was pioneered by grand entrepreneurs, such as Carl Hansmann (1886), Albin Lambotte (1909), Gerhard Küntscher (1940), and Robert Danis (1949) [15–17]. In 1958, the Arbeitsgemeinschaft für Osteosynthesefragen (AO) was founded and provided an umbrella for research, outcome documentation, education, development of implants and instrumentation [18]. As the learning curve has continued over the last decades, the surgical community has developed an improved understanding of appropriate soft tissue management. Thus, it has been realized that in patients with musculoskeletal injuries, successful management of the traumatized surrounding soft tissues plays a key role in achieving favorable surgical outcomes [19]. Although we have made rapid progress in improving surgical outcomes in patients with musculoskeletal injuries, we still have a long way to go. Despite the availability of appropriate antiseptic techniques, modern technology, and an improved understanding of soft tissue management, the risk of surgical site complications leaves significant room for improvement. As of today, the risk of surgical site infection in patients with high-risk lower extremity fractures, such as high-energy fractures of the tibial plateau, tibial plafond, and calcaneus, may be as high as 15% [20]. This rate may even be higher in elderly patients and patients with medical co-morbidities [6]. Surgical site infections continue to represent a significant healthcare and socioeconomic problem as they frequently result in hospital re-admissions, need for secondary surgeries, need for long-term antibiotics, sick leave days, disability, and patient morbidity. On a daily basis, healthcare providers make a significant effort in treating patients with surgical site infections as well as researching complex surgical strategies for salvaging musculoskeletal infections [21, 22]. While the appropriate treatment of surgical site infections remains an important effort, it remains crucial to continue our focus on preventing the actual occurrence of surgical site infections, in particular in high-risk injuries and high-risk patient populations. Thus, we must continue to explore early cost-efficient interventions that carry the potential to lower the incidence of surgical site infections. Potential surgical strategies requiring further investigation include the implementation of patient safety protocols, local antibiotic delivery systems [23, 24], and incisional negative-pressure wound therapy [6, 20]. Besides improving upon operative measures, the perioperative medical management will play an increasingly important role in the prevention of surgical site infections. As we are facing an aging patient population, we will see increasing rates of medical co-morbidities associated with poor wound healing, such as diabetes mellitus, malnutrition, and obesity. Thus, further research efforts may focus on the efficacy of perioperative medical and nutritional optimization.

As of today, there remains a critical gap of knowledge in many of these areas. For instance, we must learn more about our patients’ nutritional behaviors, diagnostic measures to identify specific nutritional deficits, and the efficacy of nutritional interventions. Over the last decades, the surgical community has made great strides in developing safe surgical techniques allowing for the successful treatment of many surgical conditions, which prior had to be left untreated. Looking forward, we must retain a strict focus on quality research and innovative strategies aimed at further minimizing the risk of postoperative wound complications as we are striving to improve surgical outcomes and patient safety in surgery.

## References

[1] Stahel PF (2018). Surgical patient safety: a case-based approach.

[2] Modi PK, Kaufman SR, Borza T, Oliphant BW, Ryan AM, Miller DC, Shahinian VB, Ellimoottil C, Hollenbeck BK. Medicare Accountable Care Organizations and Use of Potentially Low-Value Procedures. Surg Innov 2018 [Nov 30, Epub ahead of print]. 10.1177/1553350618816594 PMC6503656 30497340

[3] Stahel PF, Wang P, Hutfless S, McCarty E, Mehler PS, Osgood GM, Makary MA (2018). Surgeon practice patterns of arthroscopic partial meniscectomy for degenerative disease in the United States: a measure of low-value care. JAMA Surg.

[4] Ljungqvist O, Scott M, Fearon KC (2017). Enhanced recovery after surgery: a review. JAMA Surg..

[5] Mukherjee V, Evans L (2017). Implementation of the Surviving Sepsis Campaign guidelines. Curr Opin Crit Care.

[6] Gaunder CL, Zhao Z, Henderson C, McKinney BR, Stahel PF, Zelle BA. Wound complications after open reduction and internal fixation of tibial plateau fractures in the elderly: a multicentre study. Int Orthop 2018 [May 9, Epub ahead of print]. 10.1007/s00264-018-3940-9 29744646

[7] Scalise A, Calamita R, Tartaglione C, Pierangeli M, Bolletta E, Gioacchini M, Gesuita R, Di Benedetto G^1^Improving wound healing and preventing surgical site complications of closed surgical incisions: a possible role of incisional negative pressure wound therapy. A systematic review of the literature. Int Wound J 2016, 13(6):1260–1281. 10.1111/iwj.12492 PMC7950088 26424609

[8] Mauffrey C, Herbert B, Young H, Wilson ML, Hake M, Stahel PF (2016). The role of biofilm on orthopaedic implants: the "Holy Grail" of post-traumatic infection management?. Eur J Trauma Emerg Surg.

[9] Iqbal HJ, Ponniah N, Long S, Rath N, Kent M (2017). Review of MRSA screening and antibiotics prophylaxis in orthopaedic trauma patients; the risk of surgical site infection with inadequate antibiotic prophylaxis in patients colonized with MRSA. Injury.

[10] Lindley LE, Stojadinovic O, Pastar I, Tomic-Canic M (2016). Biology and biomarkers for wound healing. Plast Reconstr Surg.

[11] Nakayama DK (2018). Antisepsis and asepsis and how they shaped modern surgery. Am Surg.

[12] Hirsch T, Seipp H, Jacobsen F, Goertz O, Steinau H, Steinstraesser L (2010). Antiseptics in surgery. Eplasty.

[13] Miller J, Rahimi S, Lee M. History of infection control and its contributions to the development and success of brain tumor operations. Neurosurg Focus. 2005:18(4):1–5. 10.3171/foc.2005.18.4.5 15844867

[14] Tan S, Tatsumura Y (2015). Alexander Fleming (1881–1955): discovery of penicillin. Singap Med J.

[15] Hernigou P, Pariat J (2017). History of internal fixation (part 1): early developments with wires and plates before world war II. J Int Orthop.

[16] Uhthoff H, Poitras P, Backman D (2006). Internal plate fixation of fractures: short history and recent developments. J Orthop Sci.

[17] Bong M, Koval K, Egol K (2006). The history of intramedullary nailing. Bull NYU Hosp Jt Dis.

[18] Matter P (1998). History of the AO and its global effect on operative fracture treatment. Clin Orthop Relat Res.

[19] Sirkin M, Sanders R, DiPasquale T, Herscovici D Jr. A staged protocol for soft tissue management in the treatment of complex pilon fractures. J Orthop Trauma 1999, 13(2):78–84. 10.1097/00005131-199902000-00002 10052780

[20] Stannard JP, Volgas DA, McGwin G, Stewart RL, Obremskey W, Moore T, Anglen JO (2012). Incisional negative pressure wound therapy after high-risk lower extremity fractures. J Orthop Trauma.

[21] McKee MD, Li-Bland EA, Wild LM, Schemitsch EH (2010). A prospective, randomized clinical trial comparing an antibiotic-impregnated bioabsorbable bone substitute with standard antibiotic-impregnated cement beads in the treatment of chronic osteomyelitis and infected nonunion. J Orthop Trauma.

[22] McNally MA, Ferguson JY, Lau AC, Diefenbeck M, Scarborough M, Ramsden AJ, Atkins BL (2016). Single-stage treatment of chronic osteomyelitis with a new absorbable, gentamicin-loaded, calcium sulphate/hydroxyapatite biocomposite: a prospective series of 100 cases. Bone Joint J.

[23] OʼToole RV, Joshi M, Carlini AR (2017). Local antibiotic therapy to reduce infection after operative treatment of fractures at high risk of infection. a multicenter, randomized, controlled trial J Orthop Trauma.

[24] Hake ME, Young H, Hak DJ, Stahel PF, Hammerberg EM, Mauffrey C (2015). Local antibiotic therapy strategies in orthopaedic trauma: Practical tips and tricks and review of the literature. Injury.

